# The impact of a prophylactic skin dressing on surface‐guided patient positioning in chest wall Radiation Therapy

**DOI:** 10.1002/jmrs.781

**Published:** 2024-03-25

**Authors:** James Cumming, Kenton Thompson, Katrina Woodford, Vanessa Panettieri, Daniel Sapkaroski

**Affiliations:** ^1^ Department of Radiation Therapy Services Peter MacCallum Cancer Centre Melbourne Victoria Australia; ^2^ Department of Medical Imaging and Radiation Sciences Monash University Clayton Victoria Australia; ^3^ Department of Physical Sciences Peter MacCallum Cancer Centre Melbourne Victoria Australia; ^4^ Central Clinical School Monash University Melbourne Victoria Australia; ^5^ Department of Health and Biomedical Sciences Royal Melbourne Institute of Technology Bundoora Victoria Australia; ^6^ The Sir Peter MacCallum Department of Oncology The University of Melbourne Melbourne Victoria Australia

**Keywords:** Breast, chest wall, Mepitel, radiation therapy, radiotherapy, skin dressing, surface guided radiation therapy

## Abstract

**Introduction:**

Surface‐guided radiation therapy (SGRT) has emerged as a powerful tool to improve patient setup accuracy in radiation therapy (RT). Combined with the goal of increasing RT accuracy is an ongoing effort to decrease RT side effects. The application of a prophylactic skin dressing to the treatment site is a well‐documented method of reducing skin‐related side effects from RT. This paper aims to investigate whether the application of Mepitel, a prophylactic skin dressing, has an impact on the accuracy of surface‐guided patient setups in chest wall RT.

**Methods:**

A retrospective analysis of daily image‐guided Online Corrections (OLCs) from patients undergoing chest wall irradiation with SGRT was performed. Translational (superior–inferior, lateral, and anterior–posterior) OLC magnitude and direction were compared between patients treated with Mepitel applied and those treated without. Systematic and random errors were calculated and compared between groups.

**Results:**

OLCs from 275 fractions were analysed. Mean OLCs were larger for patients with Mepitel applied in the superior_inferior axis (0.34 vs. 0.22 cm, *P* = 0.049) and for the combined translational vector (0.54 vs. 0.43 cm, *P* = 0.043). Combined translational systematic error was slightly larger for patients with Mepitel applied (0.15 vs. 0.09 cm).

**Conclusion:**

Mepitel can impact the accuracy of SGRT patient‐positioning in chest wall RT. The variation however is small and unlikely to have any clinical impact if SGRT is coupled with image guidance and appropriate PTV margins. Further investigation is required to assess the effect of Mepitel on SGRT accuracy in other treatment sites, as well as any potential dosimetric impacts.

## Graphical abstract

Surface‐guided radiation therapy is used widely for chest wall radiation therapy (RT). This paper investigated whether Mepitel, a barrier film commonly used to prevent radiation dermatitis, can impact the accuracy of surface‐guided patient setups in chest wall RT by analysing image‐guided RT trends. Whilst a statistically significant impact was found, it is unlikely to be clinically significant.

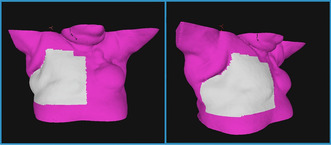

## Introduction

Accurate and consistent patient positioning is crucial to ensure radiation therapy (RT) treatments are delivered as planned at each treatment fraction. To assist in this goal, standard practice typically combines immobilisation techniques with Image‐guided radiation therapy (IGRT) technology. IGRT allows sub‐millimetre corrections to a patient's pre‐treatment position based on their internal anatomy.^1^ Surface‐guided radiation therapy (SGRT) has also emerged as a technique allowing further avenues for optimal positioning. SGRT utilises an optical tracking system to map the patient's skin surface, comparing this to a reference surface for a defined region of interest (ROI).^2^ SGRT therefore has vast applications for guiding patient setup and for monitoring inter and intra‐fraction motion. This has been demonstrated in studies that highlight the accuracy, efficiency, and safety of SGRT use in breast and chest wall radiation therapy.^3^, ^4^, ^5^

Although treatment accuracy is continuously being refined, side effects of RT remain an ever‐present concern.^6^ A common side effect is radiation dermatitis, affecting 70–99% of all patients with RT, with severity ranging from mild erythema to severe ulceration.^7^, ^8^, ^9^ Such reactions typically manifest 1–4 weeks following the commencement of treatment and will continue to develop throughout the treatment course and beyond, often requiring up to 4 weeks to properly heal.^9^ Mepitel (Mölnlycke Health Care, Gothenburg, Sweden) is a silicon‐based dressing initially designed to protect the skin following surgery and support wound healing; properties that make the film an attractive option for the management of skin reactions during RT. Several studies have shown that Mepitel film applied over the treatment area for the course of RT to the breast can reduce overall skin reaction severity by up to 92%.^10^, ^11^

Additionally, these studies found almost complete prevention of moist desquamation in breast cancer patients receiving 40–54 Gy when Mepitel film was applied during treatment. Results from studies assessing Mepitel film on head and neck RT patients receiving a higher dose (50–70 Gy) also show significant reductions in skin reaction severity and moist desquamation rates when the film is applied.^12^, ^13^, ^14^ Guided by this research, our department identified post‐mastectomy chest wall patients as the cohort to which Mepitel application would be most practical and impactful, given radiation dermatitis can be considerable in this group.^15^ Consequently, prophylactic application of the dressing has been implemented for these patients at our centre.

While Mepitel application has shown significant benefits in reducing radiation dermatitis, there is limited documentation on how the application of the dressing may influence other aspects of RT procedures, such as setup and treatment accuracy. It is possible that the application of the dressing to a patient's skin may distort the shape of the tissue beneath, particularly if the film is applied to an area of mobile tissue. Such changes in patient contour may be significant when utilising SGRT for patient setup, particularly if the film is applied during a course of treatment and is not reflected in the SGRT reference surface, potentially leading to the introduction of systematic positioning errors. Additionally, the film can be reflective once applied to the patient's surface and may interfere with the SGRT system's ability to accurately track the patient's surface, leading to difficulties with setup.

To date, there are no known studies assessing the influence of Mepitel or similar barrier films on setup accuracy when utilising SGRT. The implementation of SGRT in our clinical centre and our routine use of Mepitel on patients receiving RT to their chest wall following mastectomy has offered a unique opportunity to provide insight into this potential issue. This investigation aimed to determine if the Mepitel application can impact SGRT setup accuracy in this patient cohort by analysing daily kV IGRT correction trends. While there is less tissue to be distorted by Mepitel after mastectomy, the assessment of SGRT accuracy in these patients offers baseline insight from which further research focusing on different anatomical areas can be built upon.

## Methods

This retrospective cohort study included patients undergoing RT to their chest wall post‐mastectomy, treated consecutively at a single institution. Due to resource constraints, a 15‐month recruitment period from the SGRT introduction was implemented. To control for potential confounding variables, patients with deep inspiration breath hold (DIBH) and patients with surgical reconstructions were excluded from the study. Patients were also excluded if setup with SGRT was abandoned throughout the treatment course (i.e. due to limitations with staff or transfer to a machine without SGRT) or if kV‐image guidance was not used (i.e. when field borders were matched on skin for bilateral treatments or re‐treatments in preference of IGRT). Ethics approval, including a waiver of consent, was obtained from the Peter MacCallum Cancer Centre Human Research Ethics Committee (Ethics Project ID: QA/94287/PMCC).

### Radiation therapy CT and planning

All patients underwent computed tomography (CT) simulation with standardised immobilisation. Patients were positioned supine on the Sabella Flex™ (CDR Systems, Calgary, AB, Canada) breast board with arms raised above their heads and a located knee support under their knees. Patients with supraclavicular nodal involvement were positioned lying flat, while patients with chest wall involvement only were positioned with a 10–15° incline. A single planning CT scan was performed in free‐breathing.

Contouring and planning were carried out in Eclipse Vn. 16.0 (Varian Medical Systems, Palo Alto, CA, USA). The ‘Search Body’ segmentation tool was used to generate the body contour, with the lower threshold range set to −700 HU. Where build‐up (BU) was required, this was created as a structure in Eclipse and 3D printed for use on treatment. The BU structure was added to the patient body structure using the addition boolean operator to create an additional SGRT reference surface.

### Treatment setup with SGRT

**Figure 1:**
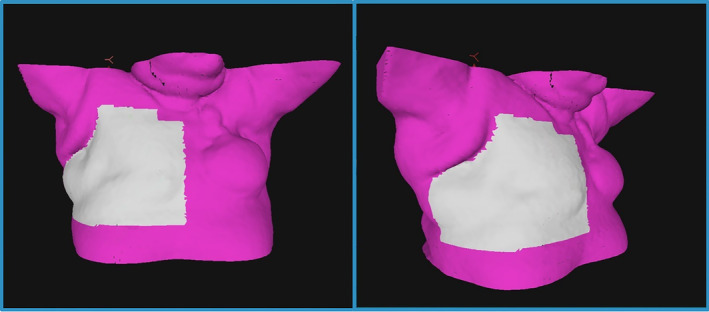
Example of standard chest wall region of interest (white). Patient contour is seen in purple. Image courtesy of VisionRT, London, UK.

**Figure 2:**
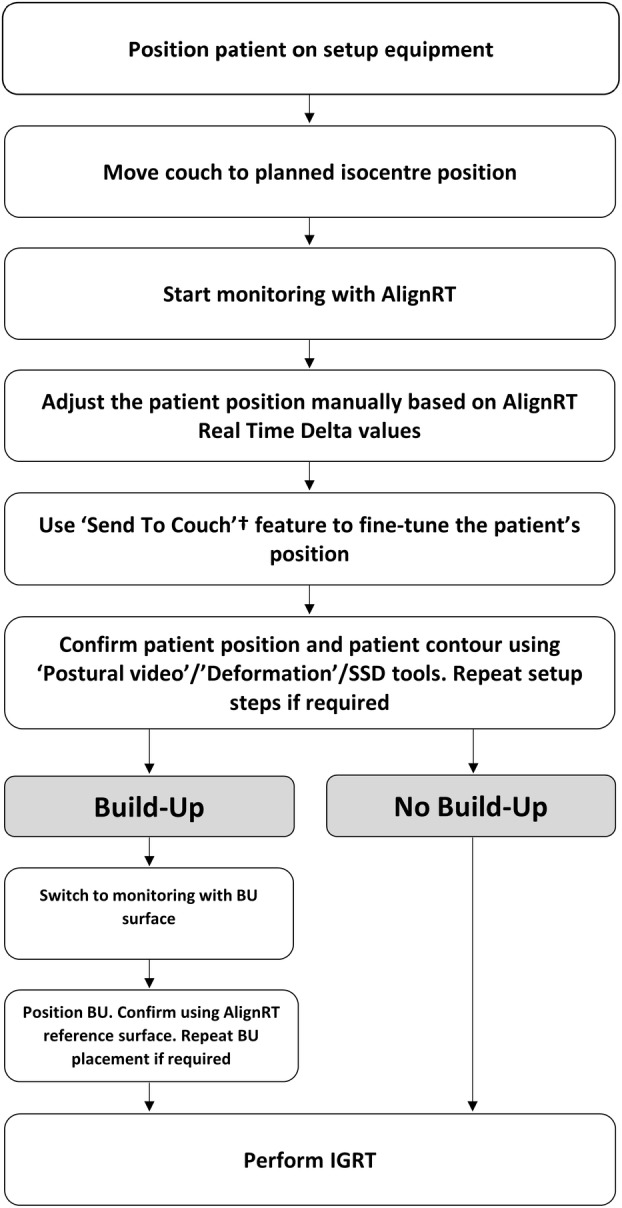
Chest wall surface‐guided radiation therapy (SGRT) free‐breathing treatment workflow with and without Build‐up (BU). †’Send to couch’: The couch is moved in all 6° automatically to bring the patient's position as close to planned as possible based on the real‐time delta values.

AlignRT Advance Vn. 6.3 (Vision RT, London, UK) was used for the setup and intra‐fraction monitoring of all patients. The planning CT surface was used as the reference surface for patient setup as default. A departmentally defined breast AlignRT protocol was used for all patients, consisting of a standard ROI definition as recommended by vendor guidelines, and standard setup and monitoring thresholds of ±0.2 cm and ±2° for translational (superior–inferior, anterior–posterior and lateral) and rotational (rotation/yaw, pitch and roll) directions respectively. The ROI included the ipsilateral chest wall from approximately midline to the posterior‐axillary line, avoiding the under‐arm, and extending longitudinally from approximately sternal notch level to the inferior level of the sternum, covering the treatment field (Fig. 1). Departmental protocol for patient setup with AlignRT was followed for all patients (Fig. 2). Real‐time delta (RTD) values representing positional displacement in each axis were used to guide initial patient setup. Once close to tolerance, the finalisation of the setup position was then performed using AlignRT's “Send to Couch” function, whereby the treatment couch is moved automatically at six degrees to match the patient's external surface with the reference surface as close as possible, effectively bringing RTD displacement values to zero. Patient contour was assessed following setup using AlignRT's “postural video”, “deformation”, and source to skin distance (SSD) measurement tools.

### Image‐guided radiation therapy

Following setup with SGRT, daily, pre‐treatment image guidance was used to finalise the patient's position. A kV‐pair comprising both a lateral and anterior or posterior kV image was taken. A bone match to the chest wall and sternum was then performed utilising translational corrections. A ‘zero action threshold’ approach was employed, whereby all required corrections greater than zero were applied. The corrections were recorded automatically within the record and verify software, MOSAIQ (Elekta AB, Stockholm, Sweden).

### Mepitel application

Mepitel was recommended based on standard departmental guidelines for post‐mastectomy patients receiving a prescription dose of 40 Gy or more. If the patient agreed to use Mepitel, a small test patch was applied overnight to assess tolerance to the film and to ensure its adhesion to the skin. If no contraindications were observed, the full dressing was applied to the patient. Care was taken to apply the dressing with the patient in a similar position to their treatment position, with the film covering the entire treatment and AlignRT ROI area where possible, avoiding under the arm and on the neck. Mepitel application was performed by trained nursing staff and was first conducted between fractions two and five of the patient's treatment course.

The Mepitel dressings were worn by the patient continuously for the remaining treatments unless not tolerated. Regular nursing appointments were provided throughout the treatment course to monitor and maintain the dressing. The date and time of application of Mepitel were recorded in the Electronic Medical Record (EMR) for all patients who had the film applied. Similarly, any maintenance or removal of Mepitel throughout the treatment course was recorded. No patients had Mepitel film applied prior to the CT simulation.

### kV image‐guided online correction data

Online corrections (OLCs), small couch shifts applied to correct patient position following setup and IGRT, were collected for every treatment fraction for each participant included in the study. OLCs were analysed both as raw values with polarity included (magnitude and direction), and as absolute values (magnitude only) to facilitate separate analysis of magnitude and directional trends. While absolute values reflect overall setup accuracy, where larger values indicate reduced setup accuracy, the raw values with direction reflect potential directional correction trends introduced by the method of the Mepitel application. In addition, OLCs were converted into a ‘vector of displacement’ (Vector_d) measure, representing the combined absolute correction magnitude for each fraction.

$$\text{Vector}\_d=\sqrt{\mathrm{Sup}\_\mathrm{Inf}\;{\mathrm{OLC}}^{2}+\mathrm{Ant}\_\text{Post}\;{\mathrm{OLC}}^{2}+\text{Lateral}\;{\mathrm{OLC}}^{2},}$$

where: Sup_Inf OLC = translational shift made in the sagittal (superior–inferior) plane, Ant_Post OLC = translational shift made in the coronal (anterior–posterior) plane, Lateral OLC = translational shift made in the transverse (lateral) plane.

Group random error (*σ*), defined as deviations from the planned treatment position that is random in magnitude and direction, was calculated from the root mean square of the standard deviation of mean OLCs for each patient. Group systematic error (∑), defined as deviations from the planned treatment position that is similar in magnitude and direction across the course of treatment, was calculated from the standard deviation of the mean OLCs for each patient.^16^

### Statistical analysis

IBM SPSS Vn 27.0 was used for all statistical analyses. Descriptive statistics were used to summarise patient characteristics and OLCs. First fraction OLCs were analysed as a control to confirm the equivalence of patient variables between groups, since at this stage, Mepitel was not present for any patient. A Shapiro–Wilks test for normality was performed on the first fraction and remaining fraction OLCs, indicating the non‐normal distribution of OLC data for each group. A Mann–Whitney *U* test was performed for analysis of differences in the first fraction OLCs between groups, while generalised estimating equations (GEEs) were used to determine the significance of differences in OLCs between groups for the remaining fractions. Given the number of fractions per patient was variable after fraction one, the GEE model was used to account for potentially correlated repeated measures for each patient.^17^ Statistical significance was assumed where *P* < 0.05.

## Results

### Participant characteristics

Between August 2021 (SGRT implementation) and December 2022, a total of 20 patients completed RT to their chest wall in Free‐Breathing and did not have surgical reconstructions. Of these, 2 were ineligible (AlignRT abandoned in favour of positioning using reference points in 1 patient, and kV imaging not used for 1 patient), leaving 18 included in the study. Of the 18 patients included, 8 (44.4%) had Mepitel applied (M+), and 10 (55.6%) did not (M−). A summary of participant demographics is outlined in Table 1.

**Table 1:** Participant demographics and data information for non‐Mepitel (M−) and Mepitel (M+) groups.

	M−	M+	Total
No. of participants	10	8	18
Mean age (years)	70.7 (54–86)	51.1 (34–79)	64.5 (34–86)
Sex	Female 9 Male 1	Female 8 Male 0	Female 17 Male 1
Length of treatment – including boosts *n* (%)			
25 + 5#	1 (10.0)	0	1 (5.6)
25#	0	2 (25.0)	2 (11.1)
16#	1 (10.0)	0	1 (5.6)
15#	7 (70.0)	6 (75.0)	13 (72.2)
10#	1 (10.0)	0	1 (5.6)
Mean	15.6	17.5	16.7
Mean body mass index (BMI)	29.0	28.3	28.5
Total OLCs included	153	122	275

### Online corrections

OLCs captured from MOSAIQ for all included participants were analysed. After exclusion of invalid data (i.e., OLCs made in error, patient re‐setup due to incorrect positioning, kVs repeated for verification following large OLC, or, for the Mepitel group, OLCs from fractions prior to Mepitel application), 275 OLCs were included for statistical analysis (M− = 153, M+ = 122).

### First fraction OLC analysis – control

No significant differences in OLC magnitude or direction were found between the M− and M+ groups at the first fraction.

### Remaining fractions analysis

Mean absolute OLCs were larger in the M+ group than the M− group in all axes, however, statistical significance was found in only the superior_inferior axis (0.34 vs. 0.23 cm, *P* = 0.049), and overall Vector_d measure (0.54 vs. 0.43 cm, *P* = 0.043).

Analysis of raw data with polarity included found no significant differences in mean OLC on any axis.

**Figure 3:**
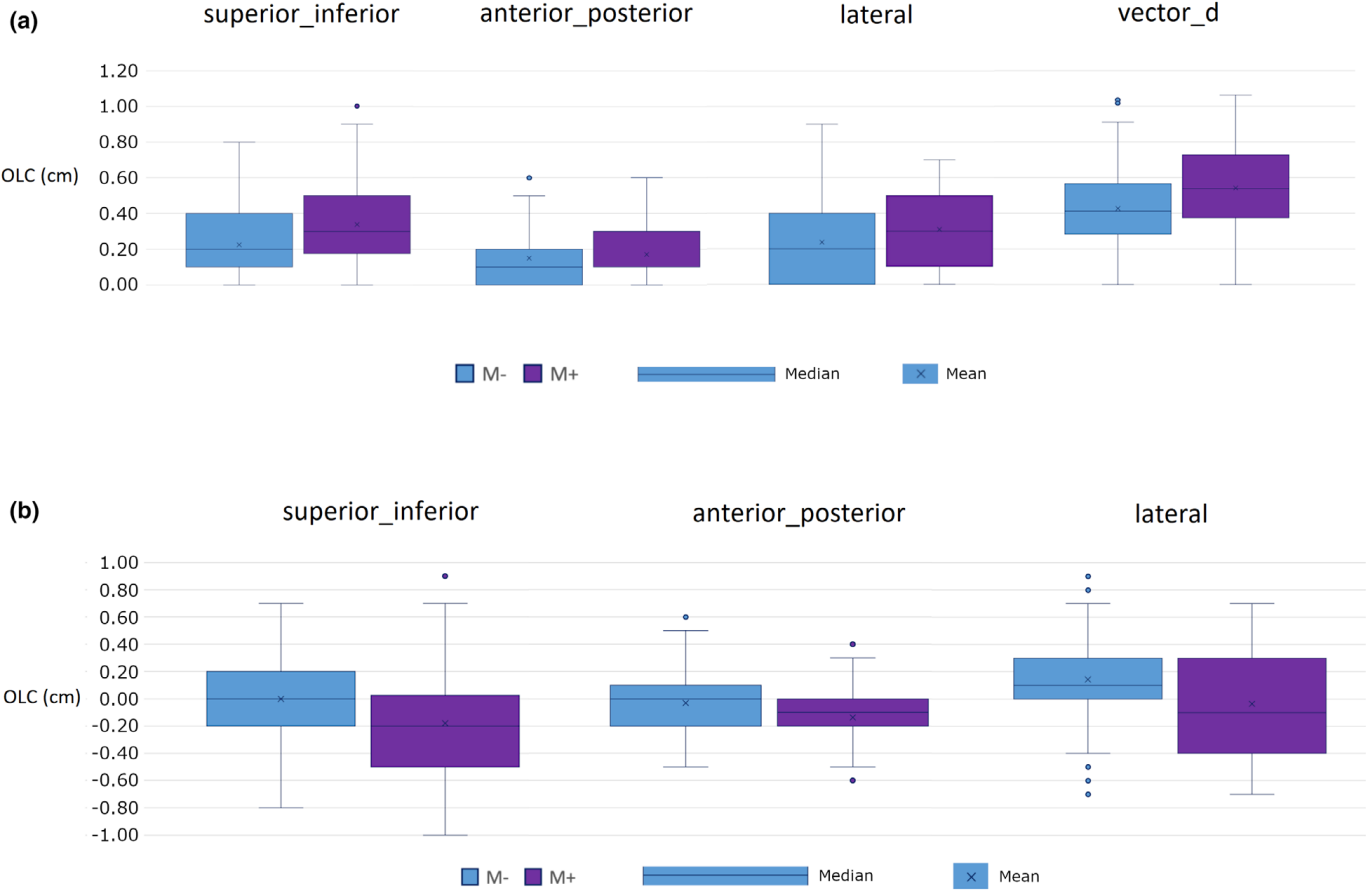
Box plots showing online correction (OLC) distribution for absolute values (a) and raw values (b) for non‐Mepitel (M−) and Mepitel (M+) groups.

A summary of mean OLC magnitudes is presented in Table 2. Box plots of OLC distribution in each axis are shown in Figure 3.

**Table 2:** Mean online correction (OLC) values for non‐Mepitel (M−) and Mepitel (M+) groups, both raw and absolute values (ABS), for each translational direction and Vector_d measure. Generalised Estimating Equation (GEE) applied for *P*‐values.

Mean OLC (cm) [Standard deviation]			
OLC Direction	M−	M+	*P*‐value
Sup_Inf			
Raw	0.00 [0.29]	−0.18 [0.37]	0.060
ABS	0.22 [0.18]	0.34 [0.23]	0.049
Ant_Post			
Raw	−0.03 [0.20]	−0.14 [0.17]	0.127
ABS	0.15 [0.14]	0.17 [0.13]	0.605
Lateral			
Raw	0.14 [0.29]	−0.04 [0.37]	0.499
ABS	0.24 [0.21]	0.31 [0.20]	0.054
Vector_d	0.43 [0.21]	0.54 [0.24]	0.043

ABS, absolute value; Ant/Post, anterior–posterior axis; M−, non‐Mepitel group; M+, Mepitel group; OLC, online correction; Sup/Inf, superior–inferior axis; Vector‐d, Vector of displacement of translational corrections.

Both random and systematic errors were greater in the M+ group in all axes except anterior_posterior, however, differences were marginal, particularly for random error. The largest increase in random error (+0.03 cm) was in both the superior_inferior and lateral axes, while the largest increase in systematic error (+0.1 cm) was in the lateral axis. Random and systematic error data are found in Table 3.

**Table 3:** Random and systematic error for non‐Mepitel (M−) and Mepitel (M+) groups.

Random (σ) and Systematic (∑) error (cm)		
OLC Direction	M−	M+
Sup_Inf		
σ	0.22	0.25
∑	0.21	0.27
Ant_Post		
σ	0.17	0.15
∑	0.11	0.11
Lateral		
σ	0.20	0.23
∑	0.21	0.31
Vector_d		
σ	0.19	0.20
∑	0.09	0.15

∑, systematic error (standard deviation of group OLC means); Ant/Post, anterior–posterior axis; M−, non‐Mepitel group; M+, Mepitel group; Sup/Inf, superior–inferior axis; Vector‐d, Vector of displacement of translational corrections; σ, Random error (root mean square of group OLC standard deviations).

## DISCUSSION

The results indicate that the introduction of Mepitel can have an impact on OLC magnitude when applied to chest wall patients and may cause a small reduction in SGRT‐guided setup accuracy. This reduction appears most prominent in the superior_inferior axis. Random error was similar between the groups, however, the slight increase in systematic error in the Mepitel group is also suggestive of overall reduced setup reproducibility following the Mepitel application. While there were statistically significant accuracy differences in the Mepitel group, they were in the order of 0.1 cm, and therefore it is unlikely that these differences would be clinically significant in the context of IGRT and the utilisation of planning target volume expansions of 0.4–0.8 cm from the clinical target volume.^18^ The small difference observed between groups is perhaps not surprising given the lack of mobile tissue typically present in this cohort of patients following mastectomy. Consequently, it is possible that the observed impact would be more prominent in patients with intact breasts or areas of treatment exhibiting more mobile tissue. Given that Mepitel is not offered to these patients routinely at our centre, a robust analysis of this patient cohort was unfortunately not possible. Therefore, it should be noted that the reported results are based on our department's specific treatment workflow for this cohort of post‐mastectomy patients. Where treatment site or other variables such as ROI definition or Mepitel application method vary from this in other departments, these results may need to be confirmed in the context of local procedures.

While the observed impact of Mepitel on SGRT setup accuracy was small, this study provides context to the sensitivity of SGRT to minor contour changes. Contour variation during RT is common in the form of weight changes and swelling, and several studies have addressed the dosimetric impact of such changes.^19^, ^20^, ^21^ Less is known however about the impact of contour changes on SGRT accuracy, and with SGRT adoption increasing worldwide,^22^ this information is relevant to a growing number of radiation oncology departments. While this study provides some context to SGRT setup accuracy following contour variation, further research is required to assess the impact of contour variation specific to weight loss, weight gain, or swelling on SGRT setup accuracy during chest wall irradiation.

In this study, the final OLC applied was based on a bony match to the chest wall and sternum, hence our analysis was comparing the difference between a surface setup to an internal anatomy image match. While we found small differences in OLCs following Mepitel application, consistency of patient position is crucial to the accurate delivery of increasingly complex RT dosimetry techniques such as intensity‐modulated RT and volumetric‐modulated arc therapy, and so even small changes in tissue shape may be detrimental to treatment quality. Petillion et al.^23^ found that employing an oblique kV imaging technique with appropriate exposure factors can adequately show the body contour on the image (along with bone, lung, and heart definition) allowing for the daily assessment of the external contour target and organs at risk while avoiding potential collision risks associated with cone‐beam computed tomography scans (CBCTs).^23^ Such a technique could further enhance confidence in treatment delivery accuracy when Mepitel is used, however further research into dosimetric impacts of contour variation is warranted.

The study's retrospective nature limits its scope, and while variables such as technique (DIBH vs FB) and surgical interventions (reconstruction vs. non‐reconstruction) were controlled, other variables like patient age and body mass index (BMI) were not. Notably, the Mepitel group was younger on average (56.8 vs. 70.7 years). Younger patients often opt for more aggressive treatments focusing on long‐term outcomes^24^ which may explain their tendency to choose to commit to the Mepitel dressing more than older patients. It is known that with increasing age, viscoelastic properties of skin change,^25^ which may lead to differences in the level of Mepitel‐induced contour distortion observed with age. However, skin elasticity at the chest is similar between the ages of 60 and 70,^26^ the approximate mean ages of each group in this study. Therefore, age‐related effects on skin elasticity are unlikely to impact the results.

Similarly, BMI was not controlled between groups. Although not a perfect indicator, higher BMIs are generally associated with increased body fat percentage^27^ which may be more prone to contour change following Mepitel application. While the mean BMI was higher for the M+ group, the difference was small (29.0 vs. 28.3) and unlikely to impact the data.

Further limitations arose from resource constraints of the retrospective study, meaning data collection was only available for a period of 15 months. While the number of patients included is small, the average course of treatment for each patient was 16.7 fractions in length. Therefore, the effective sample size was considerably more than the number of patients and still provided sufficient data for reliable analysis. Given the potential for data from individual patients to be linked, however, future studies should seek to increase patient sample size and further control for all variables such as age and BMI where possible.

While Mepitel re‐applications and modifications were recorded in the patient's EMR and documented for this study, limitations in patient numbers meant incorporating this variable into our analysis was not possible. As a result, the analysis of the impact of ongoing Mepitel maintenance was not possible, and we instead analysed only the presence of Mepitel. It would however be valuable to investigate how the maintenance of Mepitel throughout the treatment course may impact SGRT accuracy, in addition to its application. This may lead to the possibility of identifying methods of Mepitel maintenance that have minimal impact on RT setup and treatment accuracy.

## Conclusion

The application of Mepitel can impact the accuracy of SGRT patient‐positioning in chest wall RT. The variation however is small and unlikely to have any clinical impact, particularly if SGRT is coupled with IGRT and appropriate PTV margins. While limited by the retrospective nature of its design, this study provides context to the suitability and accuracy of SGRT when minor contour variation is present. Further investigation is required to assess the impact of Mepitel‐induced contour changes on SGRT accuracy in other treatment sites, including intact breasts, and the potential dosimetric impact of such changes.

## Author Contributions

James Cumming is the primary author of this study. James was responsible for the (a) conception and design, (b) completion of a review and analysis of the published literature, (c) ethics submission and correspondence, (d) data analysis interpretation, (e) manuscript drafting, and (f) provided the final approval of the version to be published. Kenton Thompson is the author of this study. Kenton was responsible for (a) clinical expertise and guidance in Radiation Therapy and Surface Guided Radiation Therapy techniques, (b) data analysis interpretation, (c) critical review of the manuscript, and (d) provided final approval of the version to be published. Katrina Woodford is the author of this study. Katrina was responsible for (a) clinical expertise and guidance in Radiation Therapy techniques, (b) data analysis interpretation, (c) critical review of the manuscript, and (d) provided the final approval of the version to be published. Vanessa Penettieri is the author of this study. Vanessa was responsible for (a) clinical expertise and guidance in Radiation Oncology Medical physics and statistical analysis, (b) data analysis interpretation, (c) critical review of the manuscript, and (d) provided the final approval of the version to be published. Daniel Sapkaroski is the supervising author of this study. Daniel was responsible for (a) completing the statistical analyses and review (b) completing a secondary review and analysis of the published literature, assisting with the interpretation of the literature reported, (c) data analysis interpretation, (d) critical review of the manuscript, and (e) provided final approval of the version to be published.

## Funding

This research did not receive internal or external funding.

## Conflict of Interest

Peter MacCallum Cancer Centre has a research agreement with VisionRT outside the submitted work. Kenton Thompson has received an honorarium and travel assistance from VisionRT outside the submitted work.

## Ethics Statement

Ethics approval for the project was granted by the Peter MacCallum Cancer Centre Human Research Ethics Committee on 17 April 2023 (Project ID: QA/94287/PMCC). This approval included a waiver of participant consent as the project meets the necessary requirements as outlined in the *National Statement on Ethical Conduct in Human Research (2018)* sections 2.3.9–2.3.12.

## Data Availability

The anonymised data that support the findings of this study are available from the corresponding author upon reasonable request.
